# K^+^-H^+^ coupling strategy for immune regulation and bone defect repair

**DOI:** 10.1016/j.mtbio.2025.101744

**Published:** 2025-04-09

**Authors:** Lintao Hu, Ke Yang, Yiyu Chen, Haoli Wang, Zezhou Fu, Lejian Jiang, Jiachen Xu, Hongsen Tian, Yiwei Zhu, Zhanqiu Dai, Yijun Li, Xianhua Chen, Xianfeng Lin, Pengfei Chen, Chenhui Gu, Shunwu Fan

**Affiliations:** a Department of Orthopaedics Surgery, Sir Run Run Shaw Hospital, Zhejiang University School of Medicine, Hangzhou, Zhejiang 310016, China; b Key Laboratory of Mechanism Research and Precision Repair of Orthopaedics Trauma and Aging Diseases of Zhejiang Province, Hangzhou, China, Hangzhou, Zhejiang 310016, China; c Department of Orthopaedics Surgery, The Fourth Affiliated Hospital of Zhejiang University School of Medicine, Yiwu, Zhejiang 322000, China; d School of Basic Medical Sciences and Forensic Medicine, Hangzhou Medical College, Hangzhou, Zhejiang 310000, China; e Department of Orthopaedics, The Second Affiliated Hospital and Yuying Children's Hospital of Wenzhou Medical University, Wenzhou, Zhejiang 325035, China; f Zhejiang Institute of Medical Device Supervition and Testing, Hangzhou, Zhejiang 310016, China; g Cixi Institute of Biomedicine, Wenzhou Medical University, Ningbo, Zhejiang 315000, China

**Keywords:** Ion homeostasis, Potassium, Intracellular pH, Immune regulation, Tissue repair

## Abstract

Ion homeostasis is crucial for maintaining cell function. Potassium ion (K^+^) is one of the most important cations in the human body, and it plays key role in maintaining biological activities and cellular functions, including the intricate balance of ion homeostasis that underpins both physiological and pathological processes. This study explored a novel role of K^+^ ions in regulating immune cell function and promoting tissue repair, especially in macrophage-mediated environments after severe tissue injury. We designed and synthesized a platelet-liposome vesicles loaded KHCO_3_ (KHCO_3_@PLV) that precisely delivered potassium bicarbonate to the site of injury extracellular after intravenous injection; then, precise ultrasound-triggered K^+^ release regulated extracellular K^+^ concentrations in the local macrophage environment. These effects collectively validate the K^+^-H^+^ coupling strategy - a novel mechanism whereby extracellular K^+^ elevation induces intracellular pH modulation, subsequently activating the AMPK/Nrf2 axis to reprogram macrophage metabolism and facilitating tissue regeneration through resolution of chronic inflammation. The main conclusion of the study is that an elevated extracellular K^+^ environment, which is an innovative treatment, is a potentially effective strategy for regulating immune responses and promoting repair after severe tissue injury.

## Highlights

• The spatiotemporal correlation between K^+^ concentration and inflammatory response in the tissue injury microenvironment.
• The development of an ion-targeted strategy involving the *in situ* release of K^+^ ions.
• Design of a K^+^-H^+^ coupling immunomodulatory therapy.

## Graphical abstract

**Figure:**
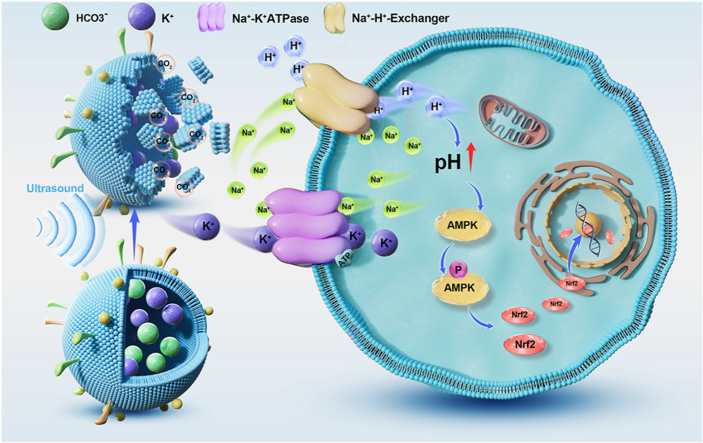

## 1 Introduction

Geologically and evolutionarily, K^+^ is importance for organisms, as the concentration gradients across cellular membranes they establish are fundamental to sustaining cellular functions and life processes. These gradients reflect the legacy of primordial environmental conditions essential for maintaining the ionic balance within and outside the cell [1,2]. Potassium ion (K^+^) play central roles in maintaining biological activities, and they are indispensable for the regulation of cell membrane potential and the maintenance of acid-base homeostasis in biological organisms. Most metabolic processes depend on or are affected by potassium [2,3]. K^+^ and H^+^ are constantly exchanged between intracellular and extracellular spaces through hydrogen-potassium exchange, which is mediated by sodium-potassium ATPase (Na^+^-K^+^-ATPase; NKA) and Na^+^-H^+^-Exchanger (NHE), to stabilize cell membrane potential and cellular pH, thereby maintaining cellular metabolism and biological functions [[4], [5], [6], [7]].

Immune cells play a crucial coordinating role in the process of tissue repair [[8], [9], [10], [11]]. In severely damaged tissues, the chronic accumulation of inflammatory macrophages can significantly delay tissue repair [12]. Therefore, it is crucial to regulate the activity of inflammatory macrophages at key time points and terminate the proinflammatory response in a timely manner to promote the effective repair of severe tissue damage. The intracellular space is characterized by hyperkalemia, and in the early stage of tissue injury, cellular rupture can release large amounts of K^+^ into the extracellular space, causing K^+^ accumulation in interstitial fluid [13,14]. The K^+^ concentration surrounding immune cells may play a role in their transformation and polarization, but K^+^ homeostasis is easily disrupted during tissue injury; therefore, we hypothesize that delayed wound healing after severe tissue injury may be related to immune dysregulation due to the extracellular K^+^ concentrations in the microenvironment.

Site of tissue injury are acidic environments, and cells need more alkaline environments to maintain their normal functions. Previous studies by our team have proposed several methods to regulate intracellular and extracellular pH; however, due to complicated cellular ion homeostasis, none of these methods can maintain a long-term pH stability [[15], [16], [17]]. Inspired by the clinical practice that K^+^ is required in addition to alkali supplementation in the treatment of patients with acidosis, we attempted to regulate the cytosolic pH at a critical time point to suppress hyperinflammation by increasing the K^+^ concentration. However, systemic increases in K^+^ concentrations carries significant risks. Inappropriate infusion of K^+^ can easily cause systemic hyperkalemia, which may lead to ventricular fibrillation or sudden cardiac arrest, severely limiting the clinical application of K^+^-related treatment approaches. During tissue repair, platelets (PLT) can accumulate at the site of injury, and the GPIb/IX/V glycoprotein complex on platelet membranes binds to the exposed von Willebrand factor (vWF) at the site of injury [18,19]. Inspired by this phenomenon, we developed a platelet-liposome vesicles (KHCO_3_@PLV) that carries potassium bicarbonate, can be intravenously injected, and precisely targets the injured tissue site.

In this study, we established a critical-size bone defect model in mice for proof-of-concept research. By comparing the metabolic characteristics of extracellular K^+^ after tissue injury and inflammatory cell phenotypes at corresponding time points, we found a correlation between the extracellular K^+^ concentrations and immune cell phenotypes. By targeting KHCO_3_@PLV to the site of injury and using noninvasive extracorporeal ultrasound stimulation, we reshaped the immune microenvironment and regulated inflammatory polarization at the critical-size bone defect site, increasing the potassium ion concentration only locally at the site of injury. By conducting transcriptomics and metabolomics analyses together, we further found that the high K^+^ concentrations in the extracellular environment regulated the inflammatory macrophage response by modulating cytosolic pH to activate the AMPK/Nrf2 signaling pathway, thus regulating excessive macrophage inflammation in the early stage of severe tissue injury and promoting effective tissue repair.

## 2 Results

### 2.1 Changes in Cell State and Ionic Environment after injury

**Fig. 1:**
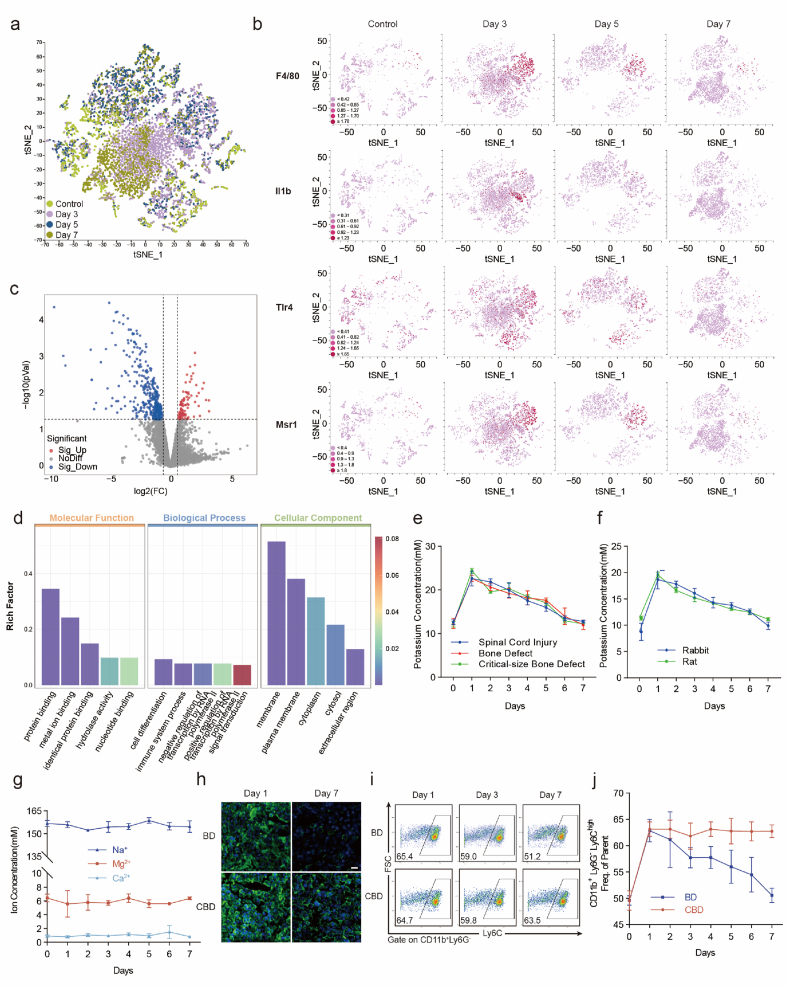
Changes in Cell State and Ionic Environment after Injury (a) Single-cell omics data of hematoma/fracture callus, populations at different time points after injured. (b) The t-SNE feature plots showed expression of four genes (*F4/80, Il1b, Tlr4 and Msr1*) over time. (c) Volcano plots of gene in macrophages of 3 day group and control group. (d) Gene ontology analysis of the top 200 genes with the greatest changes in expression. (e) Changes in K^+^ Concentrations in interstitial fluid among mice with different injuries over time (*n* = 3 independent samples, mean ± s.d.). (f) Changes in K + Concentrations in interstitial fluid among rat and rabbit over time (n = 3 independent samples, mean ± s.d.). (g) Changes in Na^+^, Mg^2+^ and Ca^2+^ Concentrations in interstitial fluid among mice over time (*n* = 3 independent samples, mean ± s.d.). (h) Immunofluorescence of iNOS at different time points in critical-size bone defect and bone defect in mice (scale bars, 25 μm). (i, j) Changes in monocyte (CD11b^+^Ly6G^−^Ly6C^high^) proportion among mice with critical-size bone defect and bone defect (*n* = 6 independent samples, mean ± s.d.).

To investigate the impact of the local microenvironment at the fracture site on cellular generation, we conducted an analysis of single-nuclei omics data from hematoma and callus collected at various time points after the fracture [20]. We employed t-distributed stochastic neighbor embedding (tSNE) to perform dimensionality reduction analysis and observed significant differences in cell grouping across different time nodes (Fig. 1a). In the t-SNE plot, we present the expression profiles of various genes (*F4/80*, *Il1b*, *Tlr4* and *Msr1*) and significant changes in macrophage proportions and function occurred over time (Fig. 1b). Macrophages were identified through rigorous screening processes by SCType, with volcano plots illustrating genes that showed differential expression (absolute log2-fold change ≥1.5, q < 0.05), followed by Gene Ontology enrichment analysis on the top 200 genes (Fig. 1c). Particularly intriguing was our observation of metal-ion binding within molecular function category (Fig. 1d). In mouse models of spinal cord injury, common bone defects, and critical-size bone defects, the K^+^ concentration substantially increased after injury and gradually decreased over time (Fig. 1e). This phenomenon was also observed in rats and rabbits (Fig. 1f). However, the concentrations of commonly occurring cations (Na^+^, Mg^2+^ and Ca^2+^) did not exhibit significant differences at the injured site (Fig. 1g). Tissue injury typically involves a significant number of cell deaths and is often followed by a period of programmed cell death [21,22]. Upon cell rupture, potassium ions are released from the cytoplasm into the interstitial fluid, resulting in a localized increase in potassium ion concentration at the injury site [23]. We examined the protein expression of iNOS in critical-size bone defects and bone defects by immunofluorescence on days 1 and 7. On day 7, iNOS expression was significantly lower in the bone defect group than in the critical-size bone defect group (Fig. 1h). Similar to the immunofluorescence results, flow cytometry revealed that the percentage of CD11b^+^Ly6G^−^Ly6C^high^ monocytes slowly decreased over time in the common bone defect group, and this phenomenon was consistent with the changes in the K^+^ concentration in the injured group. This shift is indicative of a transition from a pro-inflammatory to a more anti-inflammatory phenotype, which is instrumental in the resolution of inflammation. Nevertheless, the proportion of monocytes in the critical-size bone defect group remained high, and local inflammation could not be resolved (Fig. 1i and j). While the temporal pattern of K^+^ concentration changes is universal, the outcome of inflammation resolution is closely related to the extent of injury: in routine bone defects, the peak of K^+^ concentration coincides with the gradual resolution of the inflammatory response; however, in severe injuries such as critical-sized bone defects, despite the K^+^ concentration also experiencing an increase and subsequent decline, flow cytometry and Western blot analysis reveal that inflammation remains persistently high. This paradoxical phenomenon prompted us to hypothesize that the microenvironment of severe injuries may form a unique “imbalance in inflammatory homeostasis,” where pro-inflammatory signals dominate continuously, and repair programs are obstructed from initiating. Notably, the temporal decoupling between extracellular K^+^ concentration fluctuations and the resolution of inflammation has prompted the hypothesis advanced in this study: severe tissue injuries may be hindered in their resolution due to a relative extracellular K^+^ deficiency. This study proposes controlling excessive inflammation by maintaining elevated extracellular potassium levels via exogenous supplementation during the acute phase following severe tissue injury.

### 2.2 Extracellular K^+^ Alkalifies the Cytosol and Inhibits Inflammatory Macrophage Function

**Fig. 2:**
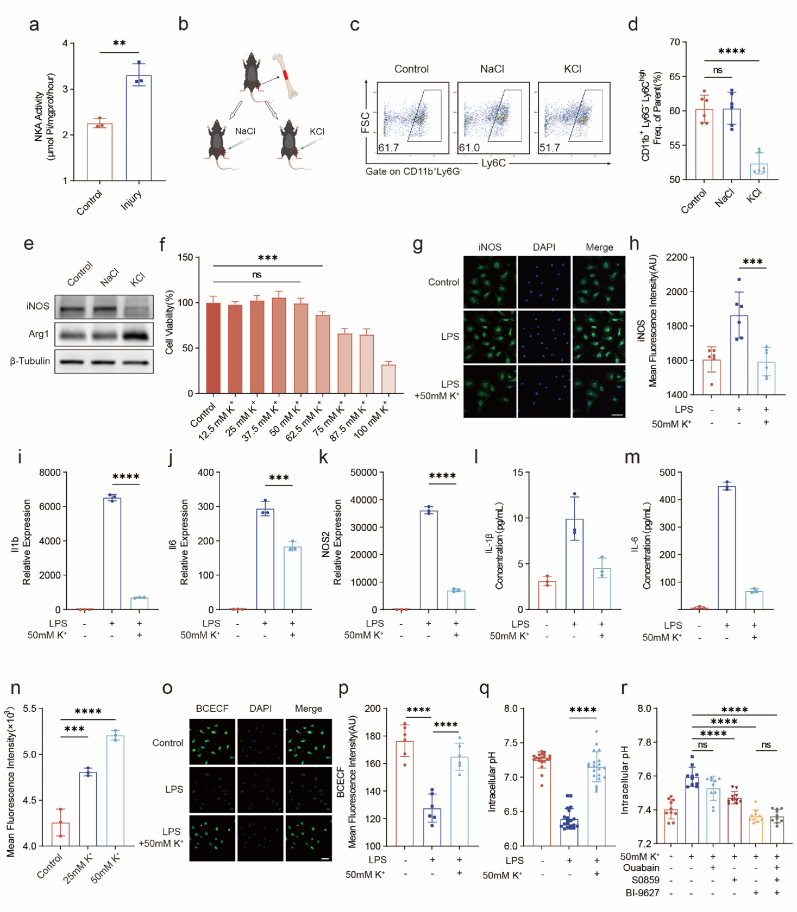
Extracellular K^+^ Alkalifies the Cytosol and Inhibits Inflammatory Macrophage Function (a) Na^+^-K^+^-ATPase activity at the injury site (*n* = 3 independent samples, mean ± s.d.). (b) Schematic diagram of KCl or NaCl solution injection (by Figdraw). (c, d) Changes in monocyte (CD11b^+^Ly6G^−^Ly6C^high^) proportion after local injection of sodium chloride or potassium chloride at the injury site (*n* = 6 independent samples, mean ± s.d.). (e) Western blot of iNOS and Arg1 after local injection of NaCl or KCl at the injury site. (f) CCK-8 assay showed the cytotoxicity of K^+^ cocultured with macrophages (*n* = 3, mean ± s.d.). (g, h) Immunofluorescence staining and quantification of iNOS levels in macrophages (scale bars, 20 μm, *n* = 6, mean ± s.d.). (i–k) Gene expression of *Il1b*, *Il6*, and *Nos2* in macrophages (*n* = 3). (l, m) ELISA of IL-1β and IL6 in macrophages (n = 3). (n) Incellular K^+^ in the indicated conditions assayed via relative fluorescence of enhanced potassium green-4 AM. (o, p) Immunofluorescence staining and quantification of BCECF-AM levels in macrophages (scale bars, 20 μm, *n* = 6, mean ± s.d.). (q) Variation of intracellular pH of macrophages (*n* = 20, mean ± s.d.). (r) Impact of various inhibitors on the modulation of intracellular pH by K^+^ (*n* = 10, mean ± s.d.).

NKA maintains a Na^+^ and K^+^ concentration gradient inside and outside the cell by depleting ATP energy and pumping Na^+^ out of the cell while pumping K^+^ into the cell. We found that the activity of NKA was significantly increased at the site of injury (Fig. 2a), and this process was closely related to the exchange of Na^+^ and K^+^ between intracellular and extracellular spaces. To verify the causal relationship between Na^+^ and K^+^ and the process of inflammatory response to the injury, we injected KCl or NaCl solution intramuscularly into the wound area (Fig. 2b) and verified the local injury-induced changes in inflammation by flow cytometry analysis of CD11b^+^Ly6G^−^Ly6C^high^ monocytes and western blotting analysis of inducible nitric oxide synthase (iNOS) and arginase 1 (Arg1) expression. An increase in the K^+^ concentration led to the resolution of inflammation (Fig. 2c–e), which further emphasizes the specificity of the potassium-mediated inflammatory resolution. Despite the potential for pain and muscle necrosis associated with local potassium chloride injections, and the risks of hyperkalemia with systemic K^+^ administration, there is an imperative need for an inflammation-targeted approach that facilitates the localized and controlled release of K^+^ ions. We redirected our focus to the significant influence of extracellular K^+^ on the intracellular environment, particularly its interaction with H^+^ in sculpting the inflammatory response. Our investigation revealed the critical role of extracellular potassium in regulating the cytosolic pH through the maintenance of ion homeostasis. This strategy, based on the K^+^-H^+^ coupling strategy, signifies a conceptual transformation in our comprehension of ion-driven immunomodulation. The cytotoxicity of extracellular K^+^ was determined by the CCK-8 method, and there was no significant inhibitory effect on bone marrow-derived macrophages (BMDMs) at the highest concentration of 50 mM K^+^ (Fig. 2f). To confirm the role of extracellular K^+^ in the polarization of BMDMs toward the M1 phenotype and the secretion of inflammatory factors, we compared the experimental group (treated with both LPS and 50 mM K^+^) with the positive control group (treated with LPS alone). These results indicated that high extracellular K^+^ concentrations significantly inhibited inflammatory gene expression. Correspondingly, immunofluorescence showed that the expression of iNOS in the LPS+50 mM K+ group was significantly lower than that in the LPS group (Fig. 2g and h). Quantitative polymerase chain reaction (qPCR) revealed that the expression of genes related to M1 macrophage function (*Il1b*, *Il6*, and *Nos2*) was significantly reduced (Fig. 2i–k). Meanwhile, enzyme linked immunosorbent assay (ELISA) showed that IL-1β and IL-6 reduced in the experimental group (Fig. 2l and m). We hypothesize that extracellular hyperkalemia regulates the pH of the cytoplasm through the biological process of hydrogen-potassium exchange, which is mediated by the combined function of NKA and NHE and involves pumping extracellular K^+^ into the cell and pumping intracellular H^+^ out of the cell. Consistent with the hypothesis about hydrogen-potassium exchange, we found that elevated extracellular K^+^ raised incellular K^+^ (Fig. 2n).During the polarization of macrophages toward the M1 phenotype after LPS stimulation, the cytoplasmic pH decreases as glycolytic acid production increases [24,25]. 2′,7′-bis-(2-carboxyethyl)-5-(and-6)-carboxyfluorescein-acetoxymethyl ester (BCECF-AM) was used to analyze the pH of the macrophages [15,16]. Using LCM, we observed a significant reduction in the fluorescence intensity of 2′,7′-bis-(2-carboxyethyl)-5-(and-6)-carboxyfluorescein (BCECF) during macrophage polarization. However, elevating extracellular K^+^ concentration markedly increased BCECF fluorescence intensity in the cytoplasm (Fig. 2o and p). By the microplate reader, increased extracellular K^+^ concentrations effectively reversed macrophage acidification during polarization (Fig. 2q). To confirm the mechanism by which extracellular hyperkalemia regulates intracellular pH, we treated cells with NKA, NHE, and sodium bicarbonate cotransporter (NBC) inhibitors. The change in cytosolic pH caused by hyperkalemia in the interstitial fluid was dominated by NHE, which is consistent with the pathophysiological theory of H^+^ and K^+^ exchange in hyperkalemia (Fig. 2r). The above characterizations indicate that changes in the extracellular K^+^ concentration can dynamically regulate the cytoplasmic pH and the polarization of macrophages through K^+^-H^+^ coupling.

### 2.3 Transcriptomics and Metabolomics Forecast Nrf2 as a Key Transcription Factor Leading to Altered Macrophages Polarization and Function

**Fig. 3:**
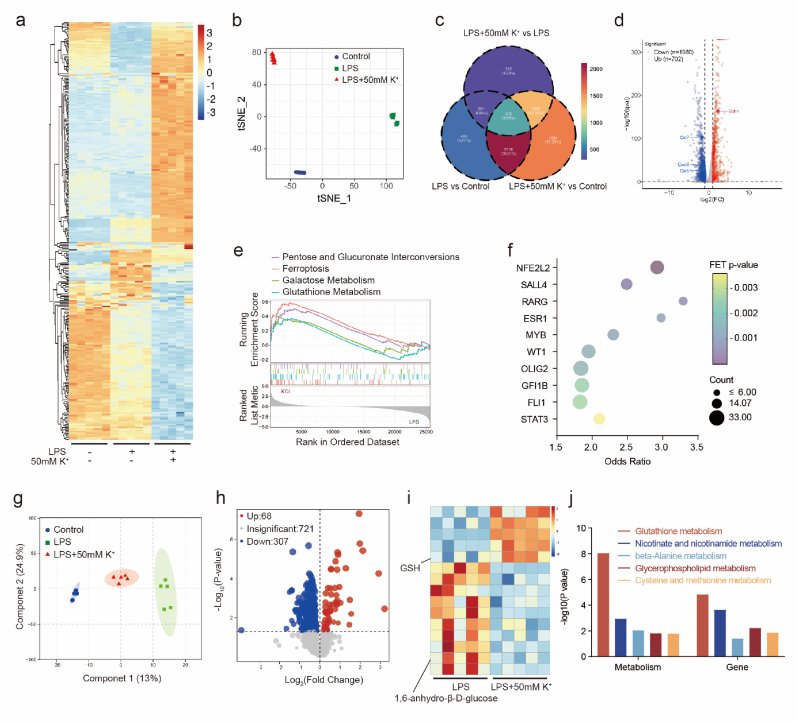
Transcriptomics and Metabolomics Forecast Nrf2 as a Key Transcription Factor Leading to Altered Macrophages Polarization and Function (a) Heatmaps of the top 200 genes with the greatest changes in expression from the three groups. (b) tSNE of Control, LPS and 50 mM K^+^-treated groups. (c) Venn diagram of total gene expression in the three groups. (d) Volcano plots of gene in macrophages after treatment with LPS or LPS+50 mM K^+^. (e) GSEA of ferroptosis, glutathione metabolism, pentose and glucuronate interconversions and galactose metabolism. (f) Analysis of the upstream regulation of differentially expressed genes after treatment with LPS or LPS+50 mM K+. (g) OPLS-DA of metabolites in macrophages after treatment with LPS or LPS+50 mM K^+^. (h) Volcano plots representing metabolites between the LPS and 50 mM K^+^-treated groups. (i) Heatmap of the metabolites between the LPS and 50 mM K^+^-treated groups. (j) Joint analysis of metabolomics and transcriptomics by KEGG.

However, there is a disparity (an order of magnitude) between the concentrations of K^+^ and H^+^. By increasing extracellular K^+^ levels, the continuous regulation of intracellular H^+^ efflux can be achieved, thereby inducing long-term cytoplasmic alkalization. To investigate the changes in intracellular transcript levels in response to increased extracellular K^+^ concentrations, we analyzed BMDMs from the LPS and LPS+50 mM K^+^ groups by transcriptome sequencing. Heatmaps show the top 200 genes with the greatest changes in expression from the three groups (Fig. 3a). The genes in the Control, LPS, and LPS+50 mM K^+^ groups were found to be highly spatially different by tSNE, constituting different subsets (Fig. 3b). The Venn diagram show 12962 differential expressed genes (absolute fold change≥ 2, q < 0.05) were identified among the three groups (Fig. 3c). A volcano plot of the differential expressed genes between LPS group and LPS+50 mM K^+^ group, revealed that 702 genes were upregulated and that 1980 genes were downregulated (Fig. 3d). Gene set enrichment analysis (GSEA) revealed that glutathione, pentose and glucuronic acid interconversion, galactose metabolism, ferroptosis and other pathways were significantly upregulated in the LPS+50 mM K^+^ group compared with the LPS group (Fig. 3e). To identify the key transcription factors that are responsible for the changes in macrophage polarization and function upon the addition of 50 mM K^+^, we selected the top 200 genes with the greatest changes in expression, constructed mRNA expression profiles of transcription factors, and used primitive pathway analysis with ChEA3 to predict upstream transcription factors. The most likely predicted transcription factor was NFE2L2 (Nrf2) (Fig. 3f). In addition, metabolomics analysis was performed, and OPLS-DA revealed that repetitive sequences clustered into distinct subsets (Fig. 3g). The volcano plot and heatmap show the differentially abundant metabolites between the LPS group and LPS+50 mM K^+^ group (Fig. 3h and i). We found that the contents of carbohydrates and their metabolites in LPS+50 mM K^+^ group were significantly lower than that in the LPS group, and reduced glutathione (GSH) was significantly increased. Joint transcriptomics and metabolomics enrichment analysis results by KEGG showed coenrichment of glutathione metabolic pathways (Fig. 3j). The intracellular content of GSH, which is a downstream metabolite of Nrf2, was measured.

### 2.4 K^+^ exerts anti-inflammatory effects by inducing cytoplasmic alkalization and activating the AMPK/Nrf2 signaling pathway

**Fig. 4:**
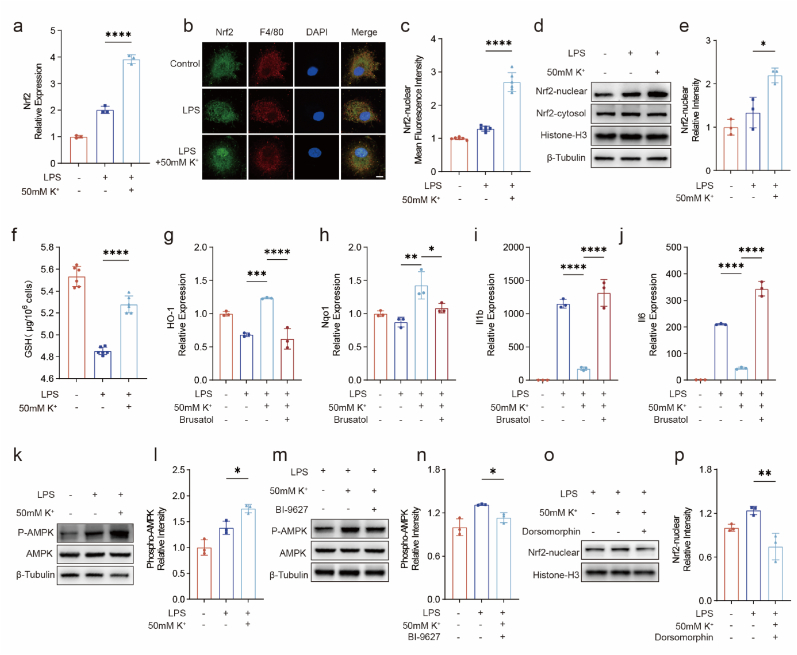
K^+^ exerts anti-inflammatory effects by inducing cytoplasmic alkalization and activating the AMPK/Nrf2 signaling pathway. (a) Gene expression of *Nrf2* in macrophages after treatment with LPS and LPS + 50 mM K^+^. (b, c) Immunofluorescence staining and quantification of Nrf2 levels in macrophages nucleus after treatment with LPS or LPS + 50 mM K^+^ (scale bars, 5 μm, *n* = 3, mean ± s.d.). (d, e) Western blot and quantification of Nrf2 levels in macrophages nucleus after treatment with LPS or LPS + 50 mM K^+^ (*n* = 3 independent samples, mean ± s.d.). (f) GSH in macrophages after treatment with LPS or LPS + 50 mM K^+^ (*n* = 6, mean ± s.d.). (g–j) Gene expression of *HO-1, Nqo1*, *Il1b* and *Il6* in macrophages after treatment with Nrf2 inhibitor (*n* = 3). (k, l) Western blot and quantification of Phospho-AMPK levels in macrophages after treatment with LPS or LPS + 50 mM K^+^ (*n* = 3 independent samples, mean ± s.d.). (m, n) Western blot and quantification of Phospho-AMPK levels in macrophages after treatment with Na^+^-H^+^-Exchanger inhibitor (*n* = 3 independent samples, mean ± s.d.). (o, p) Western blot and quantification of Nrf2 levels in macrophages nucleus after treatment with AMPK inhibitor (*n* = 3 independent samples, mean ± s.d.).

To verify the changes in Nrf2 expression in cells as well as the upstream mechanism, qPCR was performed. The results showed increased Nrf2 transcript levels in the LPS+50 mM K^+^ group (Fig. 4a). Immunofluorescence showed that Nrf2 translocation into the nucleus was significantly increased in response to hyperkalemia in the extracellular environment (Fig. 4b and c). Western blotting further confirmed that Nrf2 protein was significantly increased in the nuclei of macrophages after the extracellular K^+^ concentration was increased (Fig. 4d and e). Extracellular K^+^ significantly increased the intracellular content of GSH (Fig. 4f). The Nrf2 inhibitor brusatol (40 nM), which has no significant effect on inflammation, was used to inhibit the effect of extracellular K^+^. The qPCR results showed that brusatol reversed the inhibitory effect of extracellular K^+^ on inflammation (*Il1b* and *Il6*) reversed. The expression of the Nrf2 downstream antioxidant genes HO-1 and NQO1 also decreased to the levels of those in the LPS group (Fig. 4g–j). These findings demonstrated that by activating the transcription factor Nrf2, extracellular K^+^ inhibits inflammation in macrophages. In addition, consistent with the GSEA results, immunoblotting analysis revealed increased phosphorylation of AMPK, which is a protein upstream of Nrf2 (Fig. 4k and l). We hypothesize that extracellular hyperkalemia increases the cytosolic pH and induces AMPK phosphorylation, leading to Nrf2 nuclear accumulation. To test this hypothesis, we further used the NHE inhibitor BI-9627 and found the p-AMPK levels were significantly reduced (Fig. 2m and n). In addition, treatment with dorsomorphin, an AMPK inhibitor, significantly reduced Nrf2 nuclear accumulation (Fig. 2o and p).

### 2.5 Design of KHCO_3_@PLV for ion-targeted therapy

In the physiological context, severe injury is characterized by a relative deficiency of potassium ions, culminating in a disrupted inflammatory microenvironment. The equilibrium of the local microenvironment is thus compromised, which can impede the restoration of tissue homeostasis. We have conceptualized and engineered a novel class of nanovesicles, designed with precision targeting and on-demand release capabilities to artificially modulate the local potassium concentration, thereby providing a more conducive environment for repair.

**Fig. 5:**
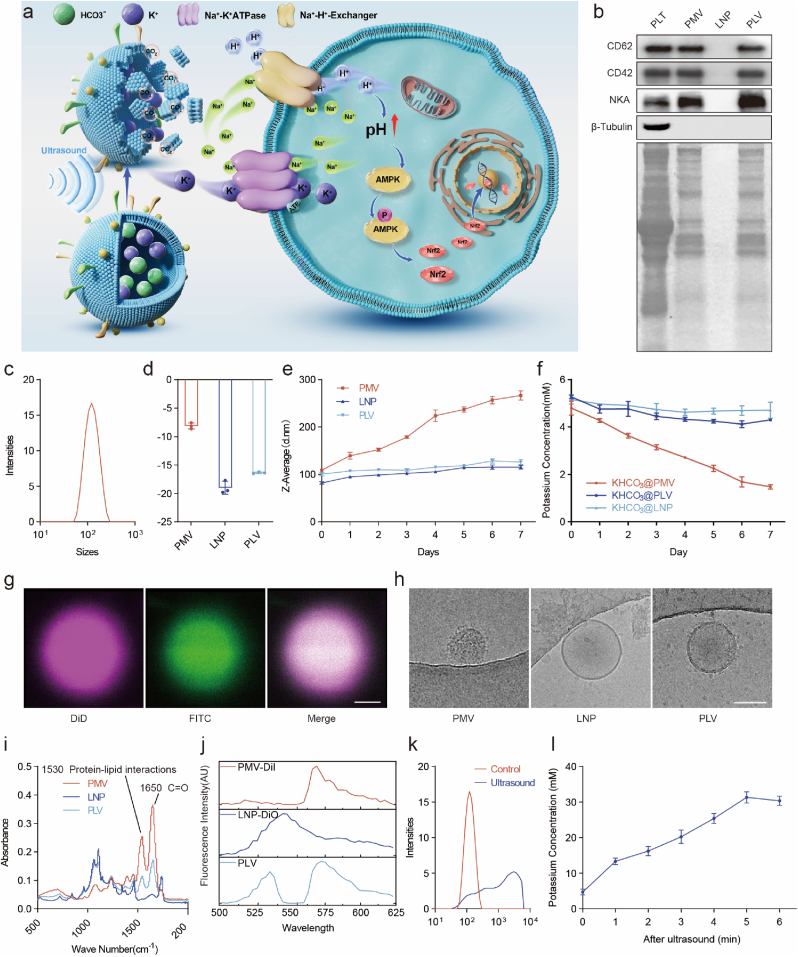
Design of KHCO_3_@PLV for Ion-targeted Therapy (a) Schematic illustration of the mechanism of KHCO_3_@PLV and its associated delivery systems. (b) Coomassie brilliant blue staining and western blot of PLT, PMV, LNP and PLV. (c) Dynamic light scattering distribution map of PLV. (d) Surface zeta potential of PLT, PMV and PLV (*n* = 3, mean ± s.d.). (e) Size changes of PLT, PMV and PLV placed in serum for different days (*n* = 3, mean ± s.d.). (f) Potassium concentration in solution of PLT, PMV and PLV placed in serum for different days (*n* = 3, mean ± s.d.). (g) LCM showed the KHCO_3_ distribution in KHCO_3_@PLV (scale bar, 1 μm) (h) Cryo-TEM images of PMV, LNP and PLV (scale bar, 100 nm). (i) FT-IR spectra of PMV, LNP, and PLV. (j) FRET of PMV (DiI), LNP (DiO), and PLV. (k) Size variation of KHCO_3_@PLV before and after ultrasound. (l) Potassium concentration in solution before and after ultrasound.

In the context of wound healing, platelets are known to aggregate at the injury site, where the GPIb/IX/V glycoprotein complex on their membrane engages with the unveiled von Willebrand factor (vWF), a critical interaction facilitating hemostasis and tissue repair [18,19]. Inspired by this native biological response, we have engineered platelet membrane nanovesicles (PMVs) from PLT, thereby harnessing the adhesive properties of platelet-derived molecules while circumventing the thrombogenic risks associated with live platelet utilization [26,27]. Subsequently, we integrated the PMV with lipid nanoparticles (LNPs) through ultrasound to fabricate a hybrid structure known as PLV (Fig. 5a).

The protein compositions of PLTs, PMVs, LNPs, and PLVs were characterized using sodium dodecyl sulfate-polyacrylamide gel electrophoresis (SDS-PAGE). Notably, significant disparities in protein composition were observed upon staining with Coomassie Brilliant Blue. PMVs and PLVs were enriched in cell membrane components, including CD42b (GPIb-IX-V), CD62 (P-Selectin), and NKA, and were devoid of intracellular components, such as β-tubulin (Fig. 5b). These findings suggest that the isolated platelet membranes were free from other cellular structures. Subsequently, the PLVs were further analyzed using nanoparticle tracking analysis (NTA) and dynamic light scattering (DLS) to determine the concentration and size distribution of KHCO_3_@PLV in solution. The concentration was determined to be 1.85 × 10^13^ particles/ml, and the mean particle size was approximately 116 nm (Fig. 5c). Additionally, the surface zeta potential of the PLVs was found between that of the PMVs and LNPs (Fig. 5d).

PMVs have inherent inflammatory targeting properties, but their stability is poor, and they are unable to maintain vesicle integrity and retain drugs. The particle size of the PLVs remained relatively stable compared to that of the PMVs, whereas the PMV particle size changed continuously over time (Fig. 5e). In addition, *in vitro* drug release experiments showed that the release rate of KHCO_3_ from the PLVs was significantly slower than that from the PMVs (Fig. 5f). Fluorescein isothiocyanate (FITC) emits a green fluorescent signal in alkaline solution [16], and KHCO_3_-FITC@PLV was prepared and stained with DiD lipid dye. Laser confocal microscopy (LCM) revealed that the KHCO_3_ solution was successfully encapsulated in the PLVs (Fig. 5g). Cryogenic transmission electron microscopy (cryo-TEM) revealed that the PMVs, LNPs and PLVs were uniformly spherical, and the PLV membrane retained many protein structures (Fig. 5h). Fourier transform infrared spectrometer (FT-IR) of PMVs, LNPs, and PLVs confirmed the presence of platelet membrane proteins in PLVs (Fig. 5i) and fluorescence resonance energy transfer (FRET) demonstrated membrane fusion of the platelet membrane and liposome (Fig. 5j). Ultrasound was used to induce the release of K^+^ in PLV *in vitro*, and the particle size was measured before and after ultrasound treatment. After ultrasound, the particles were significantly larger and no longer uniform, indicating that the vesicles were broken and reorganized under the effect of ultrasound (Fig. 5k). Meanwhile, the concentration of potassium gradually increased with the prolongation of ultrasound time (Fig. 5l).

### 2.6 Ion-targeting and release of KHCO3@PLV *In Vivo*

**Fig. 6:**
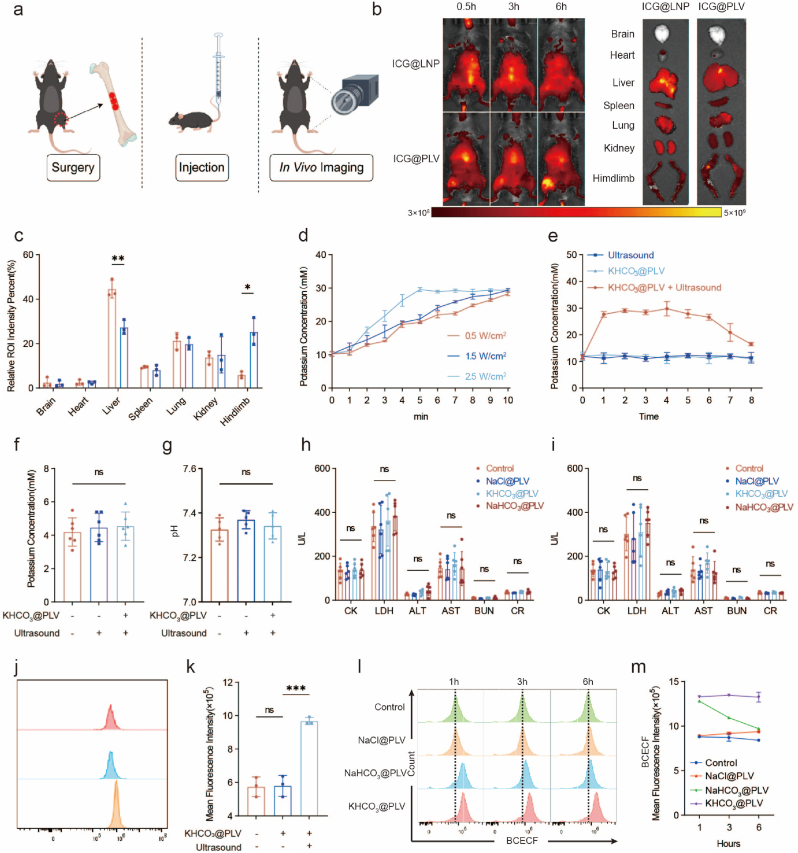
Ion-targeting and Release of KHCO_3_@PLV *In Vivo* (a) Establishment of the critical-size bone defect mice model and injection ICG@PLV (by Figdraw). (b, c) Tissue distribution of ICG@PLV and ICG@LNP in the brain, heart, liver, spleen, lung, kidney, spine and hindlimb of mice (*n* = 3 independent samples, mean ± s.d.). (d) Variations in K^+^ concentration following PLV treatment and subsequent stimulation with different ultrasound parameters. (e) With or without ultrasound release after intravenous injection, K^+^ concentration at injury site as a function of time (*n* = 3 independent samples, mean ± s.d.). (f) With or without ultrasound release after intravenous injection, the variation of serum K+ concentration (*n* = 6 independent samples, mean ± s.d.). (g) With or without ultrasound release after intravenous injection, the variation of serum pH (*n* = 5 independent samples, mean ± s.d.). (h) Heart, liver and kidney function in Control, NaCl@PLV, NaHCO_3_@PLV, KHCO_3_@PLV group on days 3 (*n* = 3 independent samples, mean ± s.d.). (i) Heart, liver and kidney function in Control, NaCl@PLV, NaHCO_3_@PLV, KHCO_3_@PLV group on days 14 (*n* = 3 independent samples, mean ± s.d.). (j, k) With or without ultrasound release after intravenous injection, the variation of incellular pH (*n* = 3 independent samples, mean ± s.d.). (l, m) Flow cytometry of the intracellular pH of cells at injury site (*n* = 3 independent samples, mean ± s.d.).

To verify that KHCO_3_@PLV can target injured sites in animals, we synthesized an ICG-encapsulated liposomes (ICG@LNP) and ICG-encapsulated platelet-liposome vesicles (ICG@PLV) using the same method. C57BL/6 mice were used to establish a femoral bone defect model, ICG@LNP and ICG@PLV (25 μL/g) were injected via the tail vein, and *in vivo* imaging was performed at 0.5 h, 3 h, and 6 h after injection (Fig. 6a).

The results showed that the fluorescence intensity at the injured site in the ICG@PLV group was significantly greater than that in the ICG@LNP group (Fig. 6b and c), and the PLVs had an obvious ability to target the injured site after intravenous injection, showing that the PLVs could accurately target the inflammatory environment at the injured site. Various ultrasound parameters were tested to optimize the rapid and complete release of K^+^ from KHCO_3_@PLV, achieving maximal ion liberation within the minimal duration (Fig. 6d). Then, KHCO_3_@PLV was injected into the mice via the tail vein, ultrasound (2.5 W/cm^2^, 60 % duty cycle) was performed at 0.5 h and 3 h after the injection, and the K^+^ concentration of the local interstitial fluid was monitored. The results showed that local K^+^ concentration was significantly elevated (Fig. 6e), while the serum K^+^ concentration and pH was not elevated (Fig. 6f and g). No adverse events or deaths were reported in any of the groups, there was no impairment of heart, liver or kidney function (ALT, AST, BUN CR, CK and LDH) or vital organs in the mice on day 3 and day 14 that were injected with NaCl@PLV, NaHCO_3_@PLV or KHCO_3_@PLV (Fig. 6h and i). We also performed H&E staining of major organs including the brain, heart, liver, spleen, lung, and kidney of mice and no abnormalities were detected (Fig. S1). We demonstrated that KHCO_3_@PLV has the potential to target sites of severe tissue damage and modulate the K^+^ microenvironment. We attempted to regulate the pH of cells at the site of injury in animals. After the critical-size bone injury model was established, the animals were injected with KHCO_3_@PLV and subjected to ultrasound. To evaluate the pH regulatory efficacy of ultrasound-triggered KHCO_3_@PLV release, pH-sensitive fluorescent probes (BCECF-AM) were employed in conjunction with flow cytometry to assess specific cell populations. The results demonstrated a significant increase in cytosolic pH following ultrasound treatment (Fig. 6j and k). The cells at injured tissues were collected at 1 h, 3 h and 6 h after ultrasound treatment, and the cytoplasmic pH was measured with BCECF-AM by flow cytometry. The results showed that the intracellular pH of the cells in the KHCO_3_@PLV and NaHCO_3_@PLV groups increased significantly at 1 h, but the pH of the cells in the NaHCO_3_@PLV group rapidly decreased at 3 h and 6 h and returned to the level of the control group at 6 h (Fig. 6l and m). We postulated that this phenomenon could be attributed to the metabolism of HCO_3_^−^ and the sustained acid production due to macrophage glycolysis. KHCO_3_@PLV has been demonstrated to increase the local K^+^ concentration, thereby regulating the local inflammatory microenvironment via K^+^-H^+^ coupling.

### 2.7 KHCO_3_@PLV Inhibits Hyperinflammation and Promotes Healing

In clinical practice, the management of severe tissue injuries typically involves a combination of pharmacological, biological, and physical therapies. Despite their efficacy, these treatments often have adverse effects, including gastrointestinal disturbances and allergic reactions. To address these limitations, we developed an ion-targeted strategy that leverages a novel K^+^-H^+^ coupling mechanism to modulate the immune response. This innovative approach effectively circumvents the common side effects associated with traditional therapies. By harnessing this mechanism, we aspire to revolutionize immunomodulation and offer a new paradigm for the treatment of severe tissue injuries, characterized by a significantly reduced side effect profile.

**Fig. 7:**
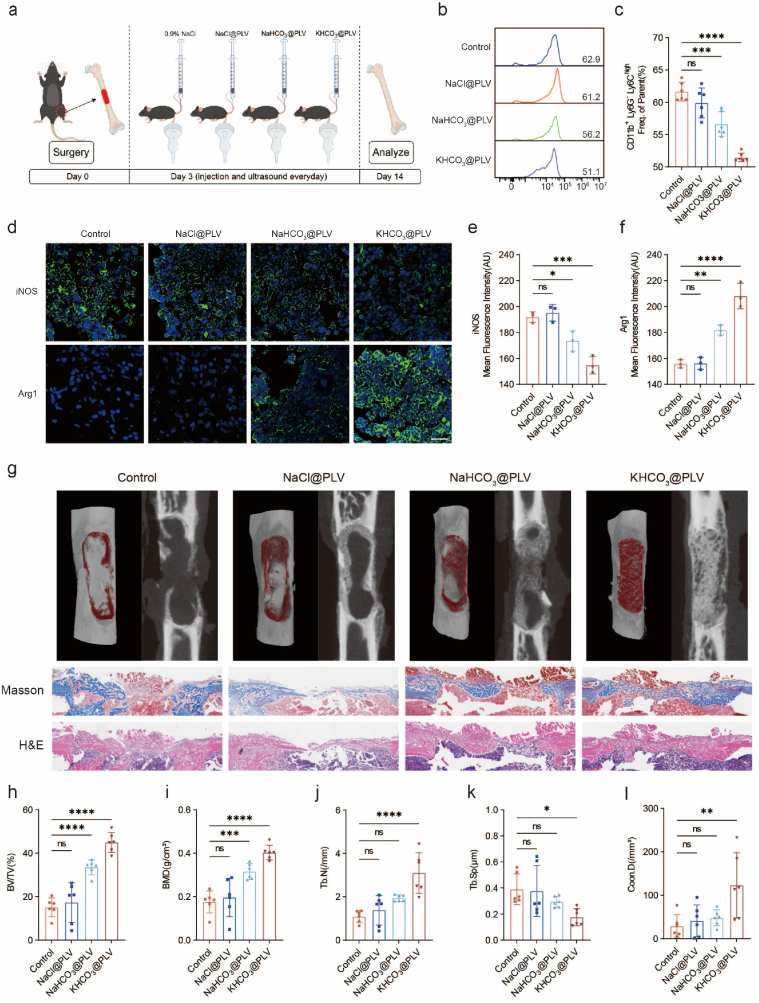
KHCO_3_@PLV Inhibits Hyperinflammation and Promotes Healing. (a) Establishment of the critical-size bone defect mice model and experimental design of the effect of KHCO_3_@PLV (by Figdraw). (b, c) Changes in monocyte (CD11b^+^Ly6G^−^Ly6C^high^) proportion after treatment of KHCO_3_@PLV (*n* = 6 independent samples, mean ± s.d.). (d–f) Immunofluorescence staining and quantification of iNOS and Arg1 of critical-size bone defect after treatment with KHCO_3_@PLV (scale bars, 20 μm, *n* = 3 independent samples, mean ± s.d.).) (g) 3D reconstruction of Micro-CT (up), Masson staining (middle) and H&E staining (down) of critical-size bone defect on days 14. (h–l) Analysis of bone mineral density and quantitative analysis of bone parameters (*n* = 6 independent samples, mean ± s.d.).

To mimic severe injury, we designed a mouse critical-size bone defect model. Ten-week-old male C57BL/6 mice were anesthetized and subjected to unilateral femoral drilling and grinding to generate a 3 mm × 1 mm bone defect. Then, the mice were randomly divided into 4 groups. On the third day after the surgery, 0.9 % NaCl, NaCl@PLV, NaHCO_3_@PLV or KHCO_3_@PLV solution were injected via the tail vein into the mice. The injured site was also subjected to ultrasound (2.5 W/cm^2^, 60 % duty cycle) for 5 min at 0.5 h and 3 h after injection (Fig. 7a). The cells from the injured site were harvested for flow cytometry on the 7th day after surgery. The proportion of inflammatory monocytes (CD11b^+^Ly6G^−^Ly6C^high^) was significantly decreased (Fig. 7b,c and Figs. S2a and b). iNOS and Arg1 are widely used as markers of pro- and anti-inflammatory macrophages, respectively. We examined the protein expression of iNOS and Arg1 by immunofluorescence on day 7. Similar to the flow cytometry results, iNOS expression was significantly decreased in the KHCO_3_@PLV group on day 7, and Arg1 expression was significantly increased on day 7 (Fig. 7d–f). KHCO_3_@PLV successfully suppressed the local inflammatory response through the K^+^-H^+^ coupling strategy. Two weeks post-surgery, the mice's femurs were harvested for micro-CT scanning, and subsequent sections were prepared for both Masson's trichrome and hematoxylin-eosin (H&E) staining. The results showed that the bone healing effect of the KHCO_3_@PLV group was obviously better than that of the control group, and the bone defect was completely covered (Fig. 7g). Quantitative analysis of bone parameters also confirmed this phenomenon. There were significant improvements in the bone volume fraction (BV/TV), bone mineral density (BMD), trabecular number (Tb.N), trabecular separation (Tb.Sp) and trabecular connection density (Conn.D) in the KHCO_3_@PLV group compared with those in the other groups (Fig. 7h–l). In particular, compared with the control group, the KHCO_3_@PLV group exhibited a significant increase in BV/TV from 15.13 % to 44.96 %, BMD from 0.18 g/cm^3^ to 0.40 g/cm^3^, Conn.D from 29.20/mm^3^ to 123.16/mm^3^, and Tb.N from 1.09 to 3.10. In addition, compared with the control and the NaCl@PLV groups, the NaHCO_3_@PLV group also showed a small improvement, which may be related to the temporary improvement in the intracellular pH.

To extend the applicability of this approach to broader tissue repair contexts, we conducted supplementary experiments by establishing a murine model of muscle injury. In muscle injury model, KHCO_3_@PLV similarly demonstrated significant anti-inflammatory effects, as confirmed through flow cytometry: the proportion of monocytes (CD11b^+^Ly6G^−^Ly6C^high^) was significantly reduced on day 3. (Figs. S3a and b). Meanwhile, we confirmed through the H&E staining on day 7 that KHCO_3_@PLV significantly alleviated excessive inflammatory infiltration (Fig. S3c). These findings robustly illustrate the therapeutic potential of localized potassium-hydrogen coupling in mitigating excessive inflammation across diverse tissue injury scenarios.

## 3 Discussion

Immune cells play a key coordinating role in the healing process of tissue injury [[28], [29], [30], [31]]. In this study, we investigated changes in the potassium concentration in interstitial fluid after severe tissue injury. Our data indicate that the decrease in potassium concentration after injury is consistent with the trend towards resolution of the inflammatory response. Spatiotemporal analysis revealed a direct correlation between the extracellular K+ concentration and the inflammatory response at the site of tissue injury. The surge in K+ postinjury, possibly due to cellular rupture, creates a high-K^+^ microenvironment that influences immune cell behavior. Our results indicate that this K^+^-rich environment may be integral to the resolution of inflammation and the promotion of tissue repair, which is consistent with the highlighted hypothesis.

The site of tissue injury is an acidic environment, and cells require a more alkaline environment to maintain normal function [28,32]. This ion homeostasis, which is based on hydrogen and potassium ions is the basis for maintaining the intracellular environment. K^+^ displaces H^+^ from the cytoplasm through hydrogen and potassium exchange to alkalinize the cytoplasm and activate AMPK phosphorylation, which promotes the nuclear accumulation of the transcription factor Nrf2. Our data elucidate the pivotal role of K^+^ in immune regulation and tissue repair, particularly in the context of severe tissue injury. The dynamic equilibrium of K^+^ and H^+^, facilitated by the interplay between Na^+^-K^+^-ATPase and the Na^+^-H^+^-Exchanger, forms the cornerstone of cellular homeostasis. Our findings underscore the importance of extracellular K^+^ in modulating the inflammatory response and its potential as a therapeutic target for tissue repair.

The K^+^-H^+^ coupling therapy developed in our study represents a paradigm shift in immunomodulation. Our innovative ion-targeted strategy involving the *in situ* release of K^+^ ions addresses the disruption of ionic homeostasis postinjury. The precision delivery system KHCO_3_@PLV allows for the controlled release of K^+^ at the injury site, thereby modulating the local inflammatory microenvironment. By harnessing the natural exchange mechanisms of cells, we engineered a system that not only corrects the ionic imbalance but also activates the AMPK/Nrf2 signalling pathway, leading to the inhibition of inflammatory macrophages and the promotion of tissue repair. This approach circumvents the risks associated with systemic K^+^ administration and offers a targeted method to regulate immune responses.

## 4 Conclusion

In this study, we present a novel paradigm for understanding inflammatory processes, elucidating the pivotal role of K^+^ in the initiation and progression of inflammation after tissue damage. Our research represents the first systematic investigation of the interplay between K^+^ and tissue damage-induced inflammation, revealing a significant correlation that has profound implications for the fields of immunology and regenerative medicine. We engineered a precision targeting strategy employing KHCO_3_@PLV to modulate the local concentration of K^+^. This approach was demonstrated to effectively stabilize the ion-immune microenvironment through a unique K^+^-H^+^ coupling strategy. The innovative nature of our approach lies in its ability to target the underlying ionic imbalances that are often overlooked in traditional anti-inflammatory treatments. In conclusion, the implications of our findings extend beyond immediate therapeutic applications. These findings provide new directions for further research into the complex relationship between ionic homeostasis and immune responses, paving the way for the development of next-generation therapies that are more efficacious and less prone to resistance.

## 5 Methods

The research reported in this article complies with all relevant ethical regulations. The animal experimental protocols were approved by the Institutional Animal Care and Use Committee of the Zhejiang Center of Laboratory Animals and followed institutional guidelines.

### 5.1 Mice and cell culture

The background of all the mice used was C57BL/6. C57BL/6 mice were purchased from Hangzhou Medical College. The animals were housed in a specific pathogen-free facility with a temperature below 25 °C, 50 % humidity and a 12 h light-dark cycle, and they were given free access to food and water. Before the mice were sacrificed, venous blood was collected from the eyes of the mice and left for 30 min. Then, the serum was collected by centrifugation at 3000 rpm for 10 min to determine heart, liver and kidney function.

BMDMs were extracted according to methods that were previously reported in the literature [15,33]. Fresh bone marrow stromal cells were isolated from 6–8-week-old C57BL/6J mice. Bone marrow cells were flushed from the tibias and femurs with cold α-MEM and cultured in α-MEM supplemented with 10 % fetal bovine serum (FBS, Gibco), 1 % penicillin/streptomycin, and 25 ng/ml macrophage colony-stimulating factor (M-CSF, R&D Systems) for 5 days to generate BMDMs. GSH content assay kit (Beijing Boxbio Science & Technology Co.,Ltd.) was used to measure GSH in macrophages.

Total RNA was extracted from the cells using AG RNAex Pro reagent (Accurate Biotechnology). Evo M-MLV RT Premix for qPCR (Accurate Biotechnology) was used for reverse transcription into cDNA. The antibodies used for WB were as follows: β-tubulin polyclonal antibody (Proteintech), AMPK alpha antibody (Affinity), phospho-AMPK alpha antibody (Affinity), and Nrf2 antibody (Affinity). To evaluate cytotoxicity, BMDMs were cultured in media supplemented with different concentrations of K^+^. After 24 h of culture, the cell viability of each group was determined with a CCK-8 kit (Yeasen).

### 5.2 Isolation of interstitial fluids

Interstitial fluid was isolated from the injury microenvironment using a validated protocol based on published protocols [34,35]. Briefly, muscle and bone were excised from the injured site, flushed with saline to remove blood from the surface, blotted gently with tissue paper to remove excess saline, and transferred to 2-ml centrifuge tubes for the isolation of interstitial fluid. The tissues were centrifuged at 50×*g* on a nylon mesh filter with 20 μm pores to remove blood and then centrifuged at 600×*g* to obtain interstitial fluid. K^+^ and Na^+^ were immediately measured by Inductively Coupled Plasma-Optical Emission Spectrometry.

### 5.3 Critical-size bone defect *In Vivo*

The anesthetic agent was a mixture of 10 mg/ml sodium pentobarbital. This solution was intraperitoneally injected at a dose of 1 ml/100 g body weight. A longitudinal lateral incision was made in the thigh to expose the femur shaft [36]. A perforated defect was drilled in the middle of the femur using a 1 mm disposable biopsy punch with a plunger (Integra Miltex). Bone defects and critical-size bone defects were defined as 1 mm × 1 mm defects and 3 mm × 1 mm defects, respectively. The surgical area was flushed with saline solution to remove the bone chips. The incision was closed in layers with sterile silk suture. For postoperative analgesia, 50 μL of 0.04 mg/ml buprenorphine hydrochloride was injected subcutaneously every 12 h for 2 days. The mice were killed 2 weeks after surgery. Femora were fully dissected and fixed in 4 % paraformaldehyde for 24 h.

### 5.4 Immune cells at the site of injury

After surgery, cells from the injured site were harvested for flow cytometry analysis. The antibodies used in FCM were as follows: FITC anti-mouse CD11b Antibody (Biolegend), Brilliant Violet 421™ anti-mouse Ly-6G Antibody (Biolegend), PE anti-mouse Ly-6C Antibody (Biolegend). The tissues sections were prepared for immunofluorescence and the antibodies were as follows: iNOS polyclonal antibody (Proteintech), Arginase-1 monoclonal antibody (Proteintech), F4/80 polyclonal antibody (Proteintech).

### 5.5 Na^+^-K^+^-ATPase activity

After the damaged tissues were homogenized in saline, a Na^+^-K^+^-ATPase activity assay kit (Elabscience® Biotechnology Co., Ltd.) was used to measure Na^+^-K^+^-ATPase activity.

### 5.6 Preparation of isolated PMV, LNP and PLV

PRP was prepared using a previously described method with minor modifications [37,38]. In brief, to prepare PMVs, PLTs were mixed in phosphate buffered saline (PBS) supplemented with 5 mM ethylenediaminetetraacetic acid (EDTA, Sigma Aldrich) and 1 mM prostaglandin e1 (PGE1, MedChemExpress). Platelet membranes were isolated by a repeated freeze-thaw process. Aliquots of platelet suspensions were first frozen at −80 °C, thawed at room temperature, and pelleted by centrifugation at 4000×*g* for 3 min. After washing with PBS solution mixed with protease inhibitor tablets, the freeze-thaw process was repeated three times, and the samples were resuspended in 150 mM KHCO_3_ solution. The protein concentration was quantified by BCA kit (Beyotime).

LNPs were prepared according to methods that were reported in the literature [15,17]. One hundred milligrams of lecithin and 16 mg of cholesterol were weighed and dissolved in 10 ml of trichloromethane, dried to a thin film in a rotary evaporator, and then shaken with 10.0 ml of 150 mM KHCO_3_ solution.

PMVs were amalgamated with LNPs at a precise protein-to-lipid mass ratio of 1:2 to facilitate synergistic therapeutic formulation. Next, ultrasonic emulsification was performed for 10 min (ultrasonication for 2 s with 3 s intervals) and then the mixture was dialyzed at 4 °C for 48 h and passed through a 0.22 μm filter to prepare the PLVs [15]. NaCl-, FITC- and ICG-loaded PLVs were prepared using a similar method.

### 5.7 Characterization of KHCO_3_@PLV

The PMV, LNP and PLV sizes and surface zeta potentials were measured by DLS using a Malvern Zetasizer Nano ZS49. The morphology was observed by cryo-TEM (200 kV, FEI Tecnai G2 F20). The particle concentration of KHCO_3_@PLV was detected by a NanoSight NS50 (Malvern Instruments Ltd.). To verify the successful fusion of the natural cell membrane and the artificial lipid membrane, membrane fusion studies were performed. The lipid membrane was labeled with a pair of FRET dyes (DiO and DiI) and fused to increasing amounts of PLT membrane [39]. Then, the overlay of the lipid membrane and PLT membrane in PLVs was visualized via confocal fluorescence microscopy when the lipid membrane and PLT membrane were labeled with the green fluorescent dye DiO (Beyotime) and the red fluorescent dye DiI (Beyotime), respectively. The protein expression levels of PLTs, PMVs, LNPs and PLVs were determined using SDS-PAGE and western blotting. Coomassie brilliant blue staining (Beyotime) was used to detect differences in protein expression among the PLTs, PMVs, LNPs and PLVs. The antibodies used in WB were as follows: ATP1A1 polyclonal antibody (Proteintech), CD62P monoclonal antibody (Proteintech), CD42b polyclonal antibody (Proteintech). The prepared PMV, LNP and PLV were added to serum at a ratio of 1:50, and the changes in particle size and remaining K^+^ concentration were measured at 0–6 days.

### 5.8 Tail vein injection and ultrasound release

After surgery, the bone defect and critical-size bone defect mouse models were injected with 0.9 % NaCl, NaCl@PLV, NaHCO_3_@PLV, or KHCO_3_@PLV solution (10 μL/g) via the tail vein. The injury site was subjected to ultrasound (2.5 W/cm^2^, 60 % duty cycle) for 5 min at 0.5 h and 3 h after injection.

### 5.9 Bone targeting *In Vivo* fluorescence imaging assay

*In vivo* IVIS spectroscopic imaging was used to confirm the bone targeting effect of PLVs [15]. PLVs encapsulated with ICG were prepared as described above. Bone defect model mice were injected with ICG@PLV solution (25 μL/g) via the tail vein. ICG biodistribution analysis was performed with a 710 nm excitation wavelength and a 785 nm filter at 0.5 h, 3 h and 6 h after injection. In addition, mouse tissues and organs, such as the spine, hind limbs, heart, liver, spleen and kidney, were collected for *in vitro* assays. The distribution of ICG@PLV was analyzed. The fluorescence intensity was quantified by Living Image software.

### 5.10 Immunohistochemistry

Following the euthanasia of the mice, the principal organs, including the heart, liver, spleen, lung, kidney, and femurs were harvested and fixed in 4 % paraformaldehyde solution for 24 h. Subsequently, these tissues sections were prepared and stained with Masson and H&E for microscopic examination.

### 5.11 Micro-CT scan and analysis

After fixation in 4 % paraformaldehyde, the femora were subjected to micro-CT scans (Bruker) with the following parameter settings: source voltage, 50 kV; source current, 450 μA; AI 0.5 mm filter; pixel size, 12 μm; object diameter, 9 mm; and rotation step, 0.2°. The obtained images were reconstructed with NRecon software using the following parameter settings: ring artifact correction, 8; smoothing, 2; beam hardening correction 30 %; and gray level correction, 0–0.14. A refined volume of interest with a height of 1 mm was generated 0.5 mm above or below the vertebral/growth plate of the distal vertebral body/femur or proximal tibia. The region of interest (ROI) for the trabecular bone within the above volume was manually defined. The constant threshold was set as 70–255, and then BV/TV, BMD and bone histomorphometry were analyzed by the CTAn (Bruker) program, utilizing the parallel plate model [[40], [41], [42]].

### 5.12 Bioinformatics data analysis

Download the single-nucleus RNAseq data of the mouse periosteal response to bone fracture from the GEO database [20], with the accession number GSE234451. Subsequently, Trailmaker platform (https://app.trailmaker.parsebiosciences.com) and OmicStudio tools (https://www.omicstudio.cn/tool) were used for data analysis. Prior to the data integration step, perform a series of quality control steps with default parameters, including Classifier filter, Cell size distribution filter, Mitochondrial content filter, Number of Genes vs UMIs filter, and Doublet filter. After these steps, proceed with data integration and opt for the tSNE algorithm in the Configure embedding for visualization analysis.

## CRediT authorship contribution statement

**Lintao Hu:** Writing – original draft, Investigation, Data curation. **Ke Yang:** Writing – original draft, Investigation, Data curation, Conceptualization. **Yiyu Chen:** Writing – original draft, Investigation, Data curation. **Haoli Wang:** Investigation, Formal analysis. **Zezhou Fu:** Visualization. **Lejian Jiang:** Validation, Investigation. **Jiachen Xu:** Visualization. **Hongsen Tian:** Formal analysis. **Yiwei Zhu:** Visualization. **Zhanqiu Dai:** Validation. **Yijun Li:** Validation. **Xianhua Chen:** Formal analysis. **Xianfeng Lin:** Funding acquisition, Conceptualization, Writing – review & editing. **Pengfei Chen:** Supervision, Methodology, Funding acquisition. **Chenhui Gu:** Writing – review & editing, Supervision. **Shunwu Fan:** Writing – review & editing, Funding acquisition, Conceptualization.

## Funding sources

The study was supported by the National Nature Science Fund of China (Grant No. 82072414, 82330077, 82322043, 82372454 and 92268113), the Zhejiang Provincial Program for the cultivation of high-level innovative health talents, “Pioneer” and “Leading Goose” R&D Program of Zhejiang (Grant No. 2024C03072, 2023C03091 and 2022C03089), 10.13039/501100001809Union Fund Project of National Natural Science Foundation of China (Grant No. U21A20351), 10.13039/501100012152China National Postdoctoral Program for Innovative Talents under Grant No. BX20240327, Ningbo Science and Technology Plan Project Key R&D Plan and “Unveiling the Leader” (2023Z194 and 2024Z211) and Hangzhou Key R&D Program, 20231203A14.

## Declaration of competing interest

The authors declare that they have no known competing financial interests or personal relationships that could have appeared to influence the work reported in this paper.

## Data Availability

Data will be made available on request.

## References

[1] Macallum A.B. (1926). The paleochemistry of the body fluids and tissues. Physiol. Rev..

[2] Mulkidjanian A.Y., Bychkov A. Yu, Dibrova D.V., Galperin M.Y., Koonin E.V. (2012). Origin of first cells at terrestrial, anoxic geothermal fields. Proc. Natl. Acad. Sci. USA..

[3] McDonough A.A., Youn J.H. (2017). Potassium homeostasis: the knowns, the unknowns, and the health benefits. Physiology.

[4] Palmer B.F. (2015). Regulation of potassium homeostasis. Clin. J. Am. Soc. Nephrol..

[5] George Chandy K., Wulff H., Beeton C., Pennington M., Gutman G.A., Cahalan M.D. (2004). K+ channels as targets for specific immunomodulation. Trends Pharmacol. Sci..

[6] Kettritz R., Loffing J. (2023). Potassium homeostasis - physiology and pharmacology in a clinical context. Pharmacol. Ther..

[7] Yellen G. (2002). The voltage-gated potassium channels and their relatives. Nature.

[8] Cooke J.P., Lai L. (2023). Transflammation in tissue regeneration and response to injury: how cell-autonomous inflammatory signaling mediates cell plasticity. Adv. Drug Deliv. Rev..

[9] Eming S.A., Murray P.J., Pearce E.J. (2021). Metabolic orchestration of the wound healing response. Cell Metab..

[10] Salhotra A., Shah H.N., Levi B., Longaker M.T. (2020). Mechanisms of bone development and repair. Nat. Rev. Mol. Cell Biol..

[11] Ono T., Takayanagi H. (2017). Osteoimmunology in bone fracture healing. Curr. Osteoporos. Rep..

[12] Wynn T.A., Vannella K.M. (2016). Macrophages in tissue repair, regeneration, and fibrosis. Immunity.

[13] Hubschmann O.R., Kornhauser D. (1983). Effects of intraparenchymal hemorrhage on extracellular cortical potassium in experimental head trauma. J. Neurosurg..

[14] Zhang X., Zhang Y., Su Q., Liu Y., Li Z., Yong V.W., Xue M. (2024). Ion Channel dysregulation following intracerebral hemorrhage. Neurosci. Bull..

[15] Tian H., Gu C., Li W., Tong T., Wang Y., Yang Y., Wang H., Dai Z., Chen P., Wang F., Lin X., Shangguan L., Wang L. (2023). Neutralization of intracellular pH homeostasis to inhibit osteoclasts based on a spatiotemporally selective delivery system. Nano Lett..

[16] Lin X., Wang Q., Gu C., Li M., Chen K., Chen P., Tang Z., Liu X., Pan H., Liu Z., Tang R., Fan S. (2020). Smart nanosacrificial layer on the bone surface prevents osteoporosis through acid–base neutralization regulated biocascade effects. J. Am. Chem. Soc..

[17] Gu C., Chen P., Tian H., Yang Y., Huang Z., Yan H., Tang C., Xiang J., Shangguan L., Pan K., Chen P., Huang Y., Liu Z., Tang R., Fan S., Lin X. (2024). Targeting initial tumour–osteoclast spatiotemporal interaction to prevent bone metastasis. Nat. Nanotechnol..

[18] Tang J., Su T., Huang K., Dinh P.-U., Wang Z., Vandergriff A., Hensley M.T., Cores J., Allen T., Li T., Sproul E., Mihalko E., Lobo L.J., Ruterbories L., Lynch A., Brown A., Caranasos T.G., Shen D., Stouffer G.A., Gu Z., Zhang J., Cheng K. (2018). Targeted repair of heart injury by stem cells fused with platelet nanovesicles. Nat. Biomed. Eng..

[19] Li X., Liu Y., Qi X., Xiao S., Xu Z., Yuan Z., Liu Q., Li H., Ma S., Liu T., Huang Y., Zhang X., Zhang X., Mao Z., Luo G., Deng J. (2022). Sensitive activatable nanoprobes for real‐time ratiometric magnetic resonance imaging of reactive oxygen species and ameliorating inflammation in vivo. Adv. Mater..

[20] Perrin S., Wotawa C.-A., Bretegnier V., Luka M., Coulpier F., Masson C., Ménager M., Colnot C. (2024). Single nuclei transcriptomics reveal the differentiation trajectories of periosteal skeletal/stem progenitor cells in bone regeneration. Elife.

[21] Roth S., Cao J., Singh V., Tiedt S., Hundeshagen G., Li T., Boehme J.D., Chauhan D., Zhu J., Ricci A., Gorka O., Asare Y., Yang J., Lopez M.S., Rehberg M., Bruder D., Zhang S., Groß O., Dichgans M., Hornung V., Liesz A. (2021). Post-injury immunosuppression and secondary infections are caused by an AIM2 inflammasome-driven signaling cascade. Immunity.

[22] Chang T.-D., Chen D., Luo J.-L., Wang Y.-M., Zhang C., Chen S.-Y., Lin Z.-Q., Zhang P.-D., Tang T.-X., Li H., Dong L.-M., Wu N., Tang Z.-H. (2024). The different paradigms of NK cell death in patients with severe trauma. Cell Death Dis..

[23] Yuan J., Ofengeim D. (2024). A guide to cell death pathways. Nat. Rev. Mol. Cell Biol..

[24] Roos A., Boron W.F., Intracellular pH. (1981). Physiol. Rev..

[25] Casey J.R., Grinstein S., Orlowski J. (2010). Sensors and regulators of intracellular pH. Nat. Rev. Mol. Cell Biol..

[26] Wang S., Duan Y., Zhang Q., Komarla A., Gong H., Gao W., Zhang L. (2020). Drug targeting via platelet membrane-coated nanoparticles. Small Struct.

[27] Dehaini D., Wei X., Fang R.H., Masson S., Angsantikul P., Luk B.T., Zhang Y., Ying M., Jiang Y., Kroll A.V., Gao W., Zhang L. (2017). Erythrocyte–platelet hybrid membrane coating for enhanced nanoparticle functionalization. Adv. Mater..

[28] Saul D., Khosla S. (2022). Fracture healing in the setting of endocrine diseases, aging, and cellular senescence. Endocr. Rev..

[29] Newman H., Shih Y.V., Varghese S. (2021). Resolution of inflammation in bone regeneration: from understandings to therapeutic applications. Biomaterials.

[30] Zheng X., Wu Y., Bi J., Huang Y., Cheng Y., Li Y., Wu Y., Cao G., Tian Z. (2022). The use of supercytokines, immunocytokines, engager cytokines, and other synthetic cytokines in immunotherapy. Cell. Mol. Immunol..

[31] Saxton R.A., Glassman C.R., Garcia K.C. (2023). Emerging principles of cytokine pharmacology and therapeutics. Nat. Rev. Drug Discov..

[32] Bahney C.S., Zondervan R.L., Allison P., Theologis A., Ashley J.W., Ahn J., Miclau T., Marcucio R.S., Hankenson K.D. (2019). Cellular biology of fracture healing. J. Orthop. Res..

[33] Yu W., Wang Z., Yu X., Zhao Y., Xie Z., Zhang K., Chi Z., Chen S., Xu T., Jiang D., Guo X., Li M., Zhang J., Fang H., Yang D., Guo Y., Yang X., Zhang X., Wu Y., Yang W., Wang D. (2022). Kir2.1-Mediated membrane potential promotes nutrient acquisition and inflammation through regulation of nutrient transporters. Nat. Commun..

[34] Mittenbühler M.J., Jedrychowski M.P., Vranken J.G.V., Sprenger H.-G., Wilensky S., Dumesic P.A., Sun Y., Tartaglia A., Bogoslavski D., A M., Xiao H., Blackmore K.A., Reddy A., Gygi S.P., Chouchani E.T., Spiegelman B.M. (2023). Isolation of extracellular fluids reveals novel secreted bioactive proteins from muscle and fat tissues. Cell Metab..

[35] Reddy A., Bozi L.H.M., Yaghi O.K., Mills E.L., Xiao H., Nicholson H.E., Paschini M., Paulo J.A., Garrity R., Laznik-Bogoslavski D., Ferreira J.C.B., Carl C.S., Sjøberg K.A., Wojtaszewski J.F.P., Jeppesen J.F., Kiens B., Gygi S.P., Richter E.A., Mathis D., Chouchani E.T. (2020). pH-gated succinate secretion regulates muscle remodeling in response to exercise. Cell.

[36] Yu X., Wan Q., Ye X., Cheng Y., Pathak J.L., Li Z. (2019). Cellular hypoxia promotes osteogenic differentiation of mesenchymal stem cells and bone defect healing via STAT3 signaling. Cell. Mol. Biol. Lett..

[37] Chen P., Pan K., Song N., Yang Y., Gu C., Zhong P., Li L., Li M., Zhang Y., Dai Z., Shangguan L., Lei C., Liu Z., Zhang J., Tang R., Liu C., Fan S., Lin X. (2023). A natural extracellular matrix hydrogel through selective nutrient restriction for hyperinflammatory starvation therapy. Matter.

[38] Dai Z., Xia C., Zhao T., Wang H., Tian H., Xu O., Zhu X., Zhang J., Chen P. (2023). Platelet-derived extracellular vesicles ameliorate intervertebral disc degeneration by alleviating mitochondrial dysfunction. Mater. Today Bio.

[39] He Y., Li R., Li H., Zhang S., Dai W., Wu Q., Jiang L., Zheng Z., Shen S., Chen X., Zhu Y., Wang J., Pang Z. Erythroliposomes (2019). Integrated hybrid nanovesicles composed of erythrocyte membranes and artificial lipid membranes for pore-forming toxin clearance. ACS Nano.

[40] Dempster D.W., Compston J.E., Drezner M.K., Glorieux F.H., Kanis J.A., Malluche H., Meunier P.J., Ott S.M., Recker R.R., Parfitt A.M. (2013). Standardized nomenclature, symbols, and units for bone histomorphometry: a 2012 update of the report of the ASBMR histomorphometry nomenclature committee. J. Bone Miner. Res..

[41] Huang Y., Dessel J.V., Depypere M., EzEldeen M., Iliescu A.A., Santos E.D., Lambrichts I., Liang X., Jacobs R. (2014). Validating cone-beam computed tomography for peri-implant bone morphometric analysis. Bone Res.

[42] Lorensen W.E., Cline H.E. (1987). Marching cubes: a high resolution 3D surface construction algorithm. Proc. 14th Annu. Conf. Comput. Graph. Interact. Tech..

