# Supplementary material for: K+-H+ coupling strategy for immune regulation and bone defect repair
Source: Mater Today Bio. 2025 Apr 9;32:101744. doi: 10.1016/j.mtbio.2025.101744 (PMC12019076; doi:10.1016/j.mtbio.2025.101744)
Supplement: Multimedia component 1 [file mmc1.docx]

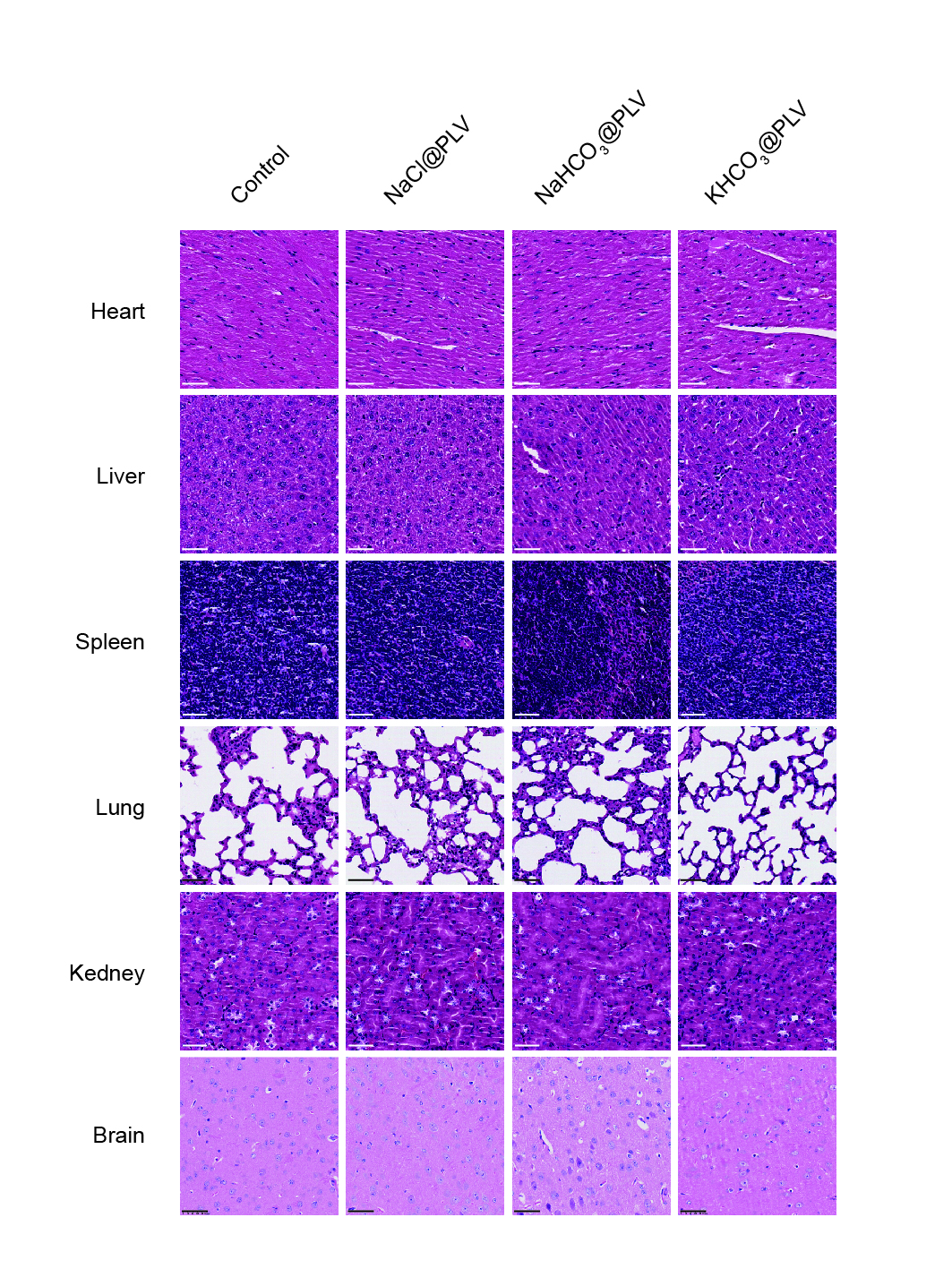


Figure S1: H&E staining of major organs following treatment with KHCO_3_@PLV.


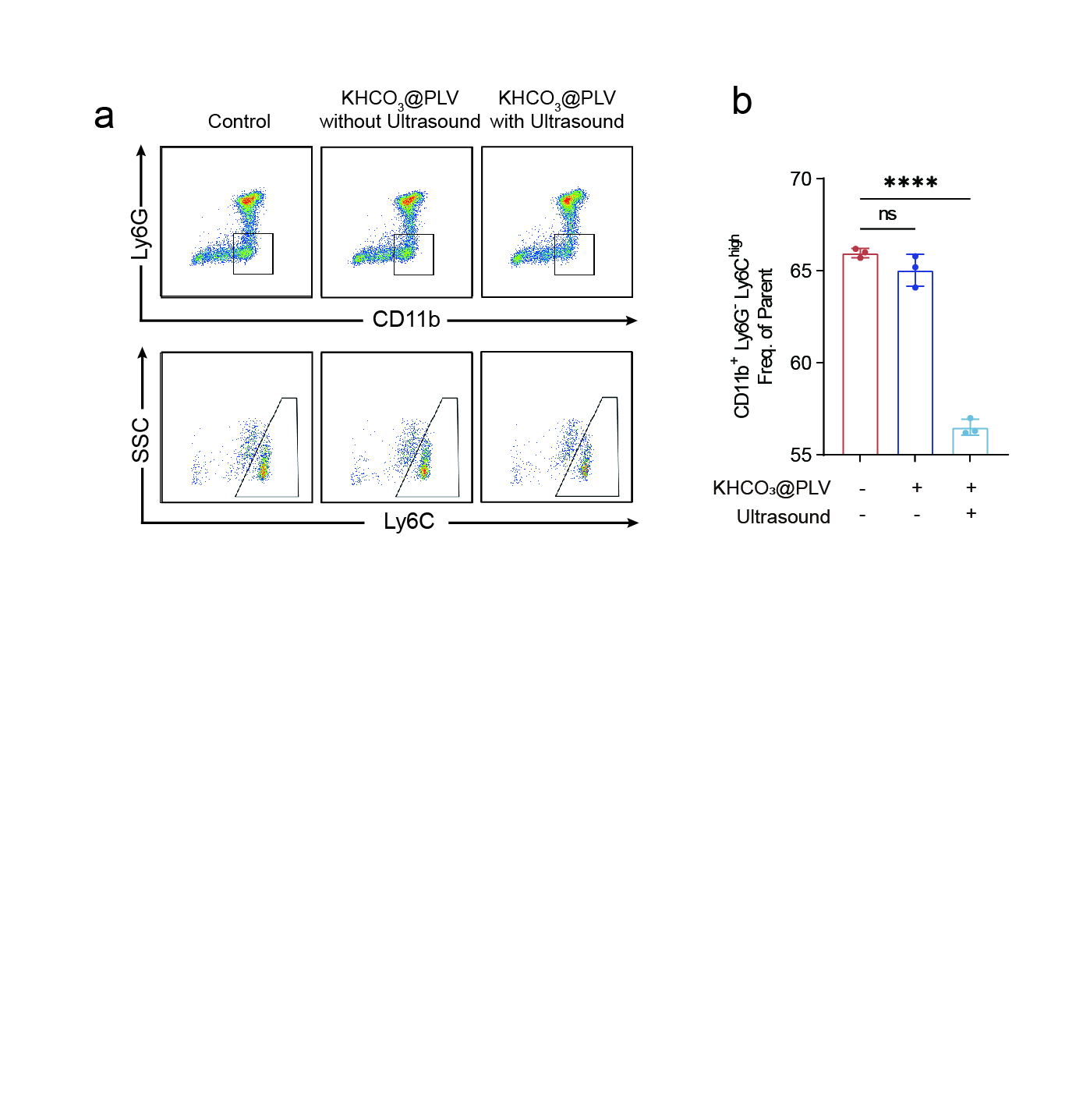


Figure S2: (a, b) Changes in monocyte (CD11b^+^Ly6G^-^Ly6C^high^) proportion after treatment of KHCO_3_@PLV with or without ultrasound (*n* = 3 independent samples, mean ± s.d.).


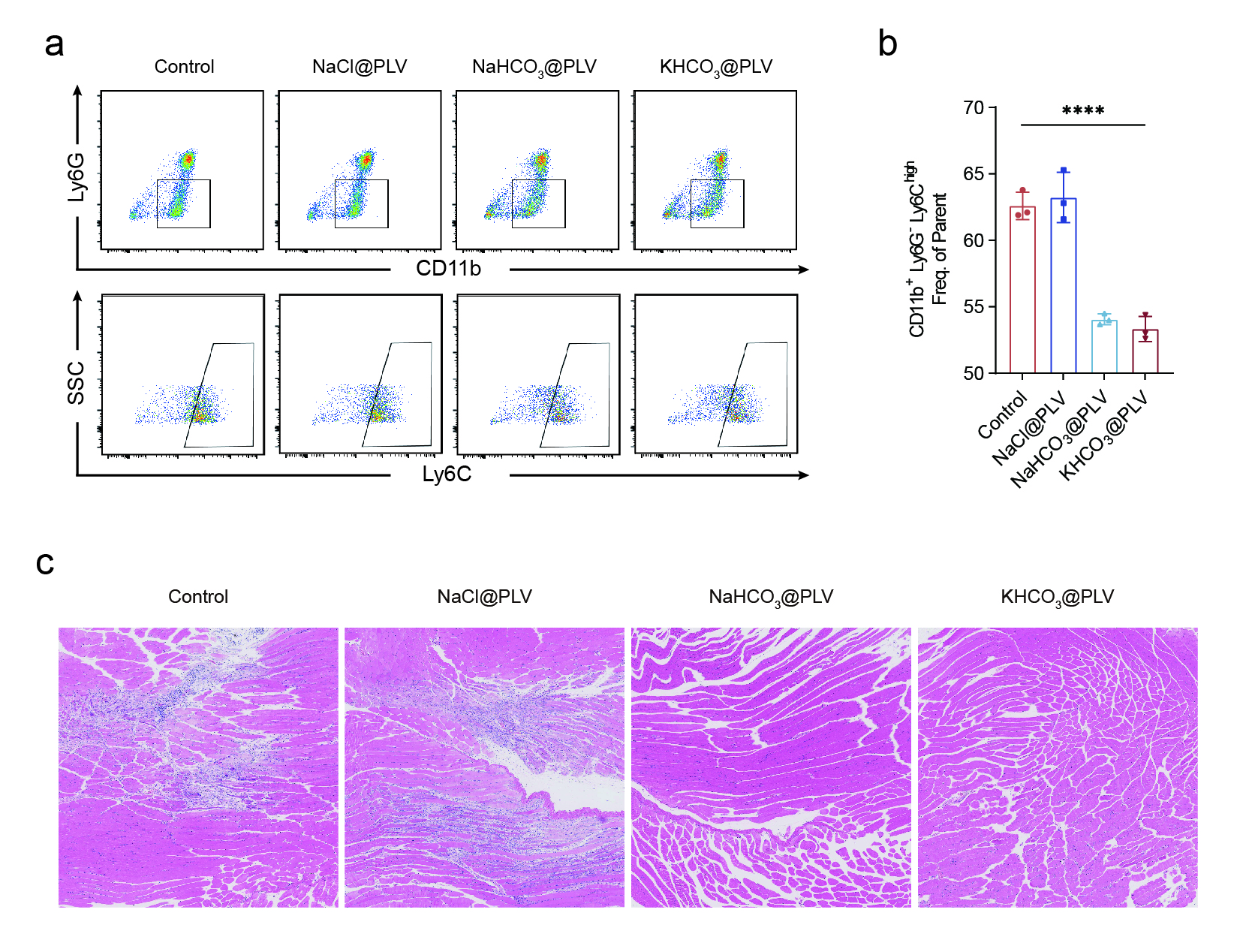


Figure S3: The role of KHCO₃@PLV in muscle injury.

(a, b) Changes in monocyte (CD11b^+^Ly6G^-^Ly6C^high^) proportion in muscle injury model after treatment of KHCO_3_@PLV (*n* = 3 independent samples, mean ± s.d.).

(c) HE staining of muscle injury on days 7.
